# Plasma proteins and mechanisms involved in the evolvement of cardiac function after myocardial infarction

**DOI:** 10.1038/s41598-026-43659-6

**Published:** 2026-03-12

**Authors:** Teun B. Petersen, Dimitris Rizopoulos, Eric Boersma, Florence Pinet, Isabella Kardys, Christophe Bauters

**Affiliations:** 1 Department of Cardiology, Thorax Center, Cardiovascular Institute, Erasmus MC, University Medical Center Rotterdam, Room Na-316, P.O. Box 2040, Rotterdam, 3000 CA the Netherlands; 2 Department of Biostatistics, Erasmus MC, University Medical Center Rotterdam, Rotterdam, the Netherlands; 3 Department of Epidemiology, Erasmus MC, University Medical Center Rotterdam, Rotterdam, the Netherlands; 4 MRC Biostatistics Unit, University of Cambridge, Cambridge, UK; 5 Inserm, CHU Lille, Institut Pasteur de Lille, U1167- RID-AGE, Université de Lille, Lille, France

**Keywords:** Heart failure, Pathophysiology, Proteomics, Biomarkers, Echocardiography, Post-MI, Biomarkers, Cardiology

## Abstract

Heart failure (HF) is a severe potential complication of myocardial infarction(MI). However precise mechanisms underlying progression from MI to HF are not yet fully understood. Exploring how plasma proteomic profiles of post-MI patients relate to cardiac function during follow-up can give insights into pathophysiological processes that contribute to HF development. We measured 4587 circulating proteins in 246 patients hospitalized for a first anterior Q-wave MI at 1, 3 and 12 months post-MI. Echocardiographic measurements of left-ventricular (LV) end-diastolic volume (EDV), LV ejection fraction (EF), and left atrial volume (AV), were assessed at hospital discharge, 3, and 12 months. Associations between protein and echocardiographic variables were assessed using pair-wise multivariate linear mixed-effects models. Median (IQR) age was 56 (46, 69) years, and 19% were women. Twenty-eight proteins were associated with LVEDV (linked to cardiac remodeling, vascular dysfunction, oxidative stress), twelve with AV (coronary artery disease, atherosclerosis, immune system), and eight with LVEF (cardiac hypertrophy, fibrosis, inflammation). Trajectories of all three echocardiographic variables were associated with NT-proBNP and BNP. Our results give an overview of the most important mechanisms related to the deterioration of cardiac function after MI, with cardiac stress, cardiac remodeling, vascular dysfunction, and inflammation emerging as central mechanisms.

**Supplementary Information:**

The online version contains supplementary material available at 10.1038/s41598-026-43659-6.

## Introduction

Myocardial infarction (MI) remains a significant health concern worldwide, associated with substantial morbidity and mortality.^1^ One of the more severe potential complications of MI is the development of heart failure (HF), which can occur in the acute phase or as a long-term consequence.^2^ The precise mechanisms underlying the progression from MI to HF are not well understood. Coronary artery disease (CAD) accounts for up to 70% of HF cases,^3^ typically presenting as HF with reduced ejection fraction (HFrEF). This is often the result of acute ischemic injury from MI and subsequent scar formation.^3^ Various animal, physiological, imaging, and clinical studies have investigated the mechanisms by which CAD leads to HF in the long term.^4^ These include microvascular dysfunction, inflammation, and remodeling. However, several aspects of these pathways remain unclear. The contribution of these different pathophysiological components to myocardial injury is likely to be heterogeneous, and a better understanding of these mechanistic pathways could help to identify novel therapeutic strategies.

Recent developments in high-throughput proteomic technologies have opened new avenues for investigating complex biological processes.^5,6^ By analyzing the temporal evolution of the plasma proteome of patients who have experienced MI and relating it to serially assessed echocardiographic measurements of ventricular function, we can uncover key pathways and biomarkers involved in the deterioration of cardiac function after MI.

In this study, we followed 246 patients who were hospitalized for MI. We serially measured 4587 circulating proteins and assessed three echocardiographic variables related to various aspects of left-ventricular (LV) and atrial function, at predetermined time-points throughout the 12-month follow-up period. Our aim was to identify protein trajectories associated with cardiac function, and to explore the underlying biological mechanisms.

## Methods

### Study population and study design

The REmodelage VEntriculaire-2 (REVE-2) study is a prospective cohort study with multicentric recruitment, which has been described in detail previously.^7^ Patients were enrolled from February 2006 to September 2008. Its research protocol was approved by the ethics committee of the “Centre Hospitalier et Universitaire de Lille” (ethical approval ID: 05/91, December 13 2005, CHRU de Lille), and written informed consent was obtained from each patient. Requalification of samples for SomaScan analysis was obtained by the “Comité de Protection des personnes Nord Ouest IV” (February 2018). Patients were included when they were hospitalized < 24 h after symptom onset and had a first anterior Q-wave MI with ≥ 3 akinetic segments at predischarge echocardiography. Exclusion criteria were: inadequate echocardiography image quality, life-limiting non-cardiac disease, significant valvular disease, or previous Q-wave MI. All methods were performed in accordance with the relevant guidelines and regulations.

### Echocardiographic measurements

Echocardiographic assessments were performed at hospital discharge (baseline = day 3 to 7), and 3 months and 12 months after initial MI. A standard echocardiographic imaging protocol was used, with apical four-chamber and two-chamber views. Two-dimensional echocardiograms of the LV short axis were recorded from the left parasternal region at three levels: the mitral valve, the mid-papillary muscle, and the apex. All echocardiograms were analyzed at the Lille Core Echo Laboratory (Lille, France), as previously described,^8^ without knowledge of the proteomic measurements. LV end-diastolic volume (LVEDV) and LV ejection fraction (LVEF) were calculated with a modified monoplane Simpson rule. Left atrial volume (AV) was calculated using the ellipsoid model as previously described.^9^ LVEDV and AV are reported as volume relative to body surface area.

### Plasma proteomic measurements

Peripheral blood samples were collected in EDTA-treated tubes in patients after MI during the initial hospitalization, and 1 month, 3 months, and 12 months after initial MI, and stored at − 80 °C. The acute phase of MI is characterized by a significant systemic inflammatory response and cellular necrosis. This can cause large, and often transient, fluctuations in a wide range of circulating proteins that may not be reflective of the longer-term pathophysiological processes driving chronic cardiac remodeling. Protein measurements at baseline were therefore not considered for the current investigation. Plasma protein levels were measured batchwise using the aptamer-based SomaScan platform (version V4.0).^10^ SOMAscan uses single stranded DNA-based protein affinity reagents called SOMAmers (Slow Off-rate Modified Aptamers), which bind to proteins with high specificity and affinity, and slow dissociation rates, to minimize nonspecific binding interactions. The results of the SOMAscan assay are provided in normalized relative fluorescent units, that are directly proportional to the amount of target protein in the initial sample. Blood samples were assayed according to the manufacturer’s protocol, and standard processes for normalization, calibration, and quality control were used as previously described.^11^.

### Statistical methods

Baseline characteristics are presented as counts and percentages for categorical variables and as means and standard deviations (SD) for continuous variables. Trajectories of the echocardiographic variables over time were modeled using linear mixed-effects models (LME), utilizing natural splines in both the fixed and random parts. Differences between echocardiographic variable measurements at hospital discharge and subsequent measurements at three months and one year were assessed using paired t tests. Correlation between mean values of serially measured echocardiographic variables per patient, and mean values of log-transformed NT-proBNP and hs-troponin T levels per patient, were visualized with scatter plots and quantified using Spearman correlation coefficients. NT-proBNP and hs-troponin T were chosen for this visualization, since they are commonly used in clinical care for patients with CAD and HF.

The pairwise association between the trajectories of the echocardiographic variables and the log-transformed protein levels was assessed by modeling them jointly via multivariate LME models, where their random effects can be correlated, and subsequently testing whether this correlation differs significantly from zero using a likelihood ratio test (false discovery rate (FDR) < 0.05). Both types of models were adjusted for age and sex. The random effect structure of time within the echocardiographic variable and protein models was specified using natural splines with two degrees of freedom with boundary knots at minimum and maximum measurement times and an interior knot at the median, iteratively dropping in complexity to random slopes or random intercepts when the full multivariate model was unable to converge. Expression in the heart and potential druggability of the associated proteins were examined using the Human Protein Atlas.^12^ As random effects involving splines do not provide a readily available interpretation for the direction of the association, we visualize this via the Spearman correlation between within-patient means of the echocardiographic variables and log-transformed protein levels of the associated proteins. The sets of proteins associated with AV, LVEDV and LVEF were analyzed for associations with biological processes of the ‘Gene Ontology: Biological Processes’ database by an enrichment analysis conducted using the Toppgene suite, with the full set of 4587 proteins taken as reference.^13,14^.

R version 4.4.1 was used for all analyses. The multivariate LME models were fitted using the nlme R package (version 3.1-168), and natural splines were incorporated using the splines R package (version 4.4.1). Tests relating the trajectories of the protein levels and echocardiographic variables were corrected for multiple testing using the Benjamini–Hochberg procedure, and considered significant when FDR < 0.05. Considerations on the statistical power are presented in the Supplemental Methods.

## Results

### Baseline characteristics

Table 1 illustrates the baseline characteristics of the cohort. The median [interquartile range (IQR)] age was 56 [46, 69] years, and 19% (46/246) were women. A total of 41% of patients had multivessel CAD, and 86% of patients underwent percutaneous coronary intervention (PCI) during hospitalization. At hospital discharge, median [IQR] LVEF was 49 [44, 55]%, while AV and LVEDV were 19.8 [16.2, 24.0] and 51 [41, 60] mL/m^2^, respectively.

**Table 1:** Baseline characteristics REVE-2.

Characteristic	*N*	*N* = 246^a^
Age (years)	246	56 (46, 69)
Women	246	46 (19%)
BMI (kg/m^2^)	246	26.9 (24.2, 29.6)
History of hyperlipidemia	246	82 (33%)
History of hypertension	246	89 (36%)
Smoking	246	168 (68%)
Diabetes mellitus	246	51 (21%)
Initial reperfusion therapy	246	
None		31 (13%)
Thrombolysis		87 (35%)
Primary PCI		128 (52%)
Multivessel CAD	237	98 (41%)
PCI during hospitalization	246	212 (86%)
Final TIMI grade 3 flow in infarct-related vessel	235	213 (91%)
Systolic blood pressure (mmHg)	239	110 (100, 120)
Diastolic blood pressure (mmHg)	239	60 (55, 70)
Heart rate (bpm)	241	69 (62, 80)
Killip class ≥ 2	246	79 (32%)
*Echocardiogram at baseline*		
Atrial volume (mL/m^2^)	229	20 (16, 24)
End-systolic volume (mL/m^2^)	244	25 (20, 31)
End-diastolic volume (mL/m^2^)	244	51 (41, 60)
Left ventricular ejection fraction (%)	244	49 (44, 55)
Wall motion score index	240	1.94 (1.81, 2.00)
*Medication*		
Antiplatelet therapy (aspirin/clopidogrel)	245	245 (100%)
β-blockers	245	238 (97%)
ACE-I or ARB	245	240 (98%)
Aldosterone antagonists	245	80 (33%)
Statins	245	231 (94%)

^a^Median (IQR); n (%). *BMI* body mass index, *PCI* percutaneous coronary intervention, *CAD* coronary artery disease, *ACE-I* angiotensin-converting enzyme inhibitors, *ARB* angiotensin receptor blockers.

### Evolution of LV and atrial function

**Fig. 1:**
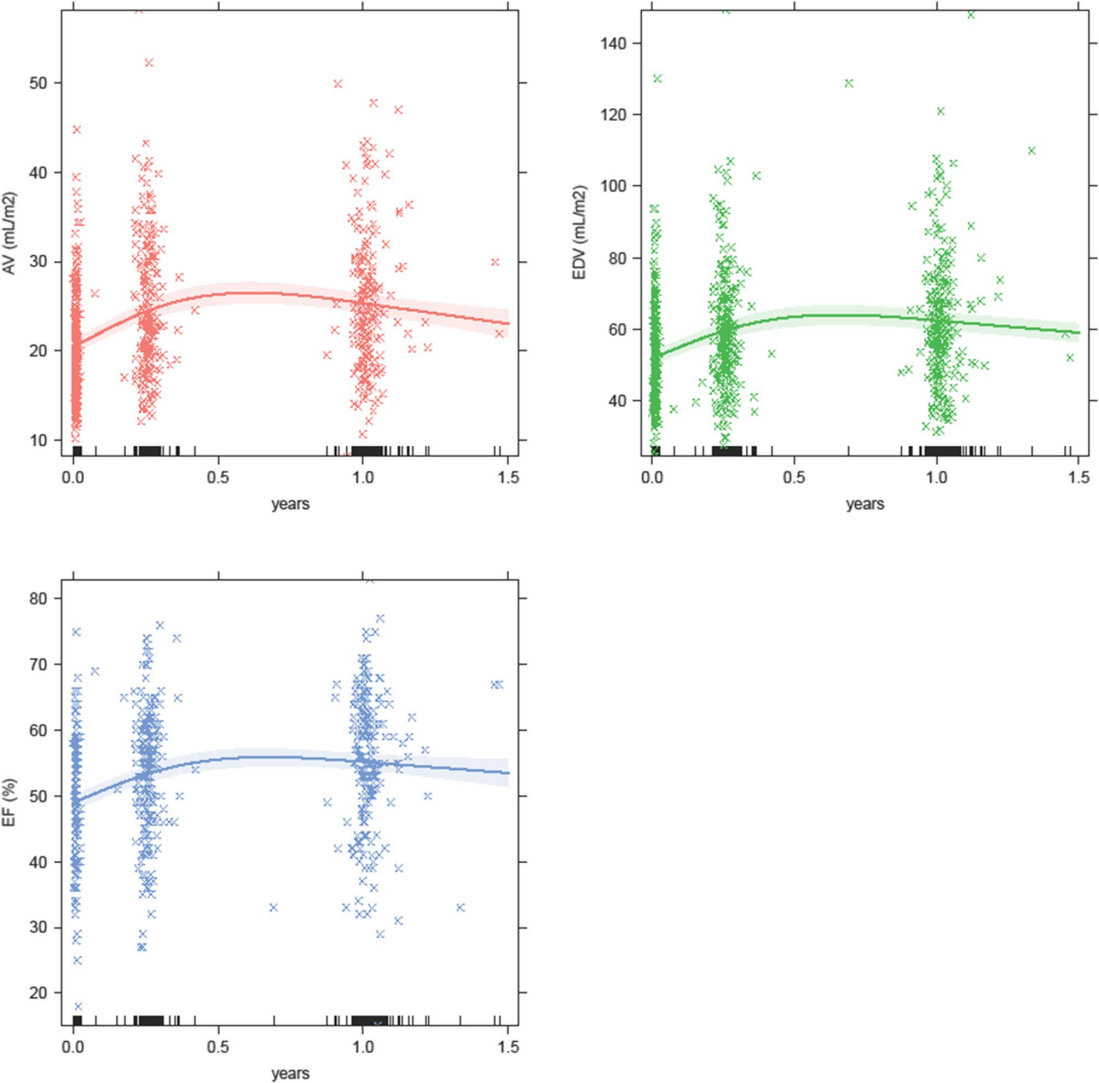
Temporal trajectories of echocardiographic variables. Population wide trajectories of atrial volume (AV; red), left-ventricular end-diastolic volume (LVEDV; green) and left-ventricular ejection fraction (LVEF; blue), as estimated via linear mixed effect models, using natural splines for the trend over time.

Figure 1 illustrates trajectories of the echocardiographic variables over time. Compared to hospital discharge, dimensions worsened over time. The modelled mean ± SD AV increased from 20.5 ± 5.9 mL/m^2^ at hospital discharge to 24.6 ± 7.4, and 25.4 ± 7.6 at three months and one year respectively, and mean ± SD LVEDV went from 52.3 ± 14.0 mL/m^2^ at hospital discharge to 59.4 ± 16.6 after 3 months and 62.3 ± 18.4 after 1 year. Conversely, LVEF showed improvement over time, as it increased from 49.3 ± 8.4% at hospital discharge to 53.9 ± 9.1% after 3 months and 55.2 ± 10.2% after 1 year (all paired t test p values < 0.001). Supplemental Table 1 displays the availability of echocardiographic variables and circulating proteins over time.

### Proteins associated with LV and atrial function

**Fig. 2:**
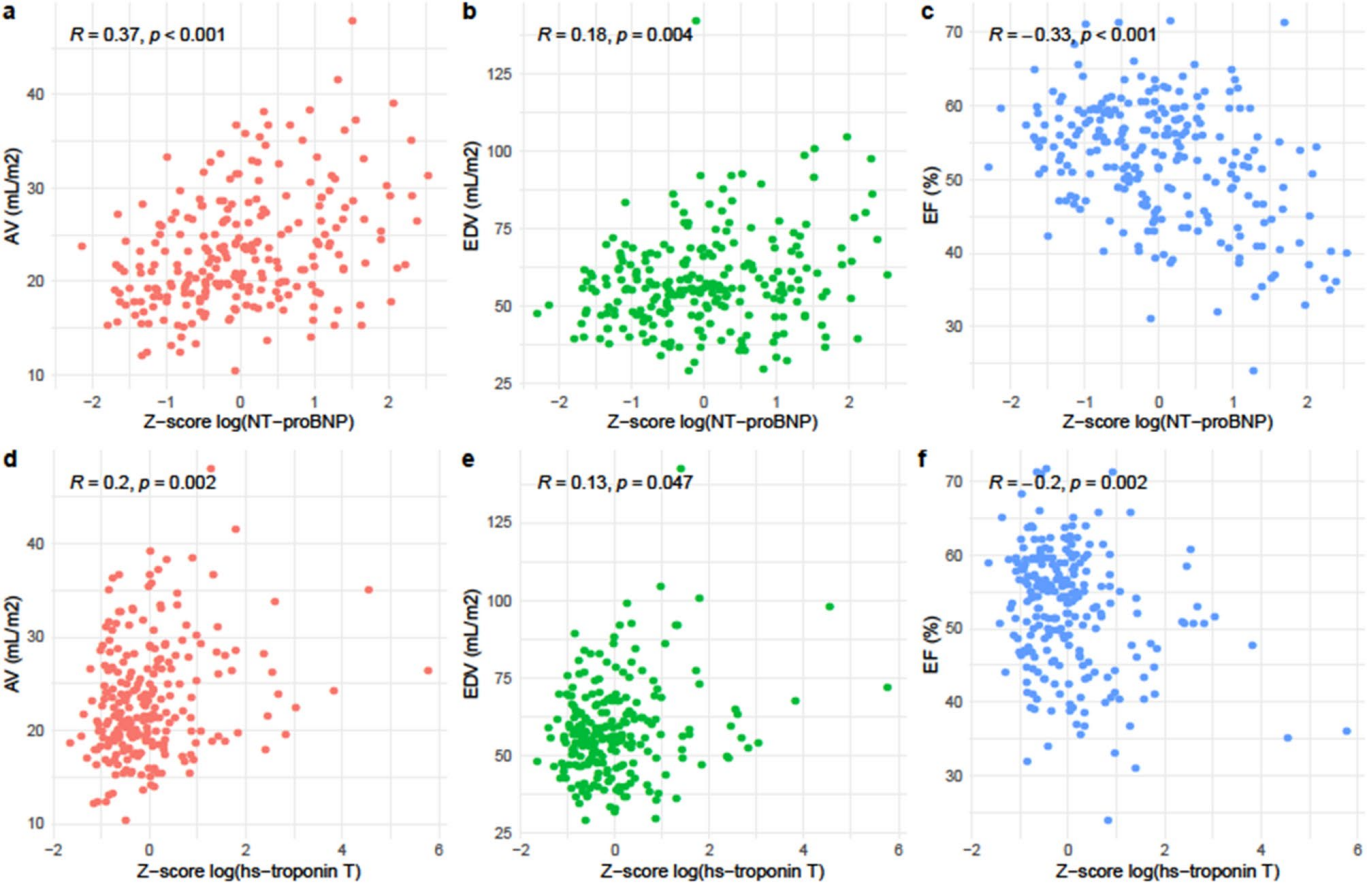
Correlation of mean values of serially measured echocardiographic variables per patient and mean values of NT-proBNP & hs-troponin T per patient. Scatter plot of within-patient mean echocardiographic variable measurements, and Z-scores of mean log-transformed NT-proBNP and hs-troponin T levels, with annotated Spearman correlation.

Figure 2 shows how mean values of serially measured echocardiographic variables per patient relate to mean values of NT-proBNP and hs-troponin T levels per patient. AV and LVEDV were positively correlated with NT-proBNP (*R* = 0.37, *p* < 0.001 and *R* = 0.18, *p* = 0.004, respectively)) and hs-troponin T (*R* = 0.20, *p* = 0.002 and *R* = 0.13, *p* = 0.047, respectively), while LVEF showed negative correlation with both proteins (*R* = − 0.33, *p* < 0.001 and *R* = − 0.20, *p* = 0.002, respectively). Although these correlations were statistically significant, their magnitude was modest. Numerically, correlations of echocardiographic variables were somewhat stronger for NT-proBNP than for hs-Troponin T.

**Fig. 3:**
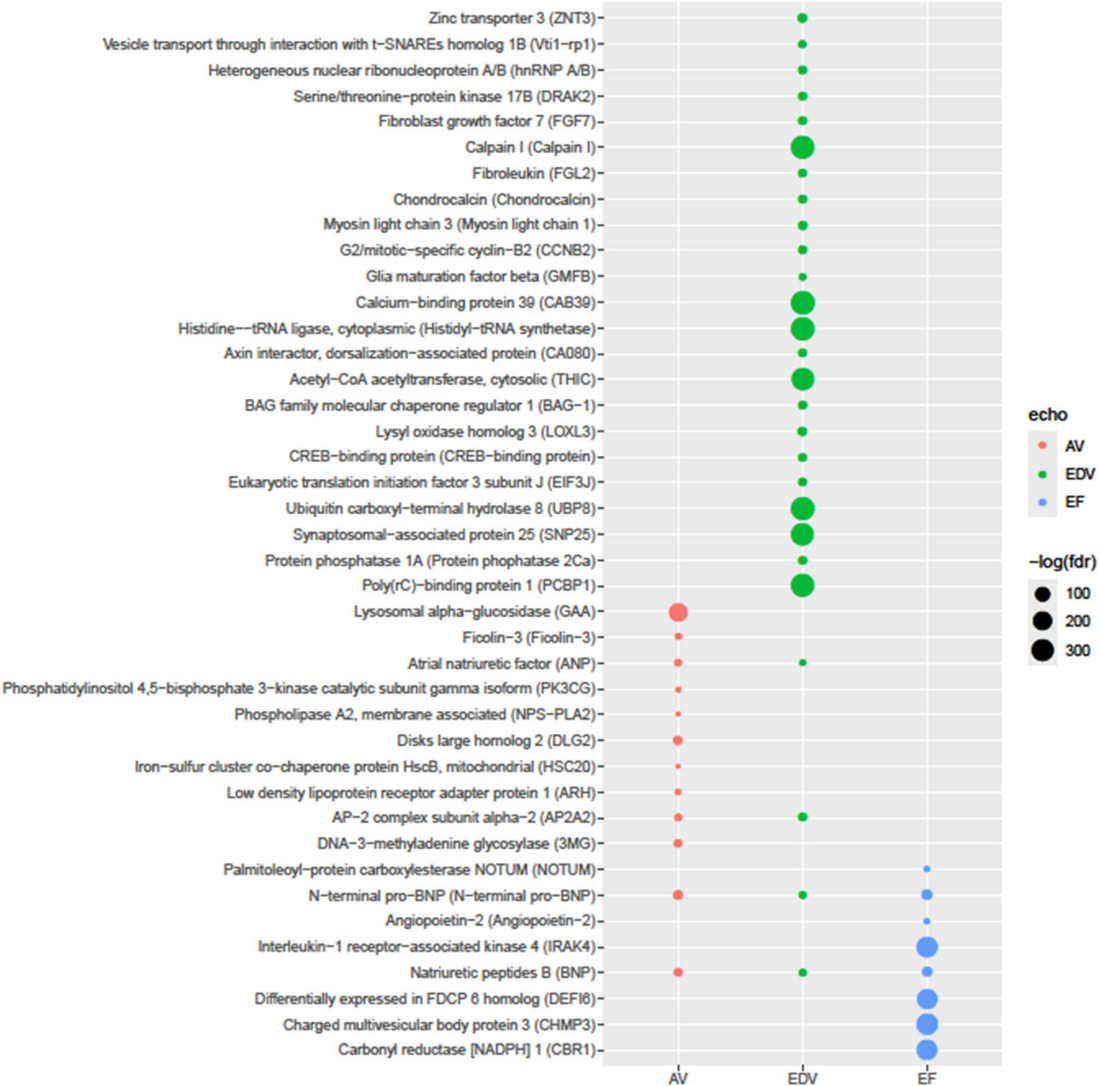
Plasma proteins associated with echocardiographic variables. Temporal trajectories of plasma proteins associated with those of the echocardiographic variables, as determined using pairwise multivariate linear mixed effect models. Only significant associations (FDR < 0.05) are shown.

Table 2 and Fig. 3 display the proteins whose trajectories were significantly associated with at least one of the echocardiographic variables. A total of 28 proteins were significantly associated with LVEDV, 12 proteins with AV and 8 with LVEF (FDR < 0.05). The top three proteins associated with LVEDV were calcium-binding protein 39 (CAB39), histidine-tRNA ligase (HARS) and ubiquitin carboxyl-terminal hydrolase 8 (UBP8). For AV these were lysosomal alpha-glucosidase (GAA), NT-proBNP and disks large homolog 2 (DLG2), and for LVEF these were charged multivesicular body protein 3 (VPS24), interleukin-1 receptor-associated kinase 4 (IRAK4) and carbonyl reductase 1 (CBR1). Moreover, the trajectories of all three echocardiographic variables were associated with NT-proBNP and brain natriuretic peptide (BNP). Additionally, AV and LVEDV shared associations with atrial natriuretic factor (ANP) and AP-2 complex subunit alpha-2 (AP2A2). Details on the convergence of the multivariate mixed effect models can be found in Supplemental Table 2.

**Table 2:** Proteins with trajectories associated with echocardiographic variables.

	Protein	FDR	Random effect structure		Correlation means^a^	Expression in heart^b^	Druggable target^b^
			Protein	Echo			
AV	Lysosomal alpha-glucosidase	1.28E−81	RI	RI	0.14 [0.01 to 0.28]	Yes	FDA approved
	N-terminal pro-BNP	5.12E−11	RI + splines	RI + slope	0.37 [0.24 to 0.47]	Elevated	FDA approved
	Disks large homolog 2	3.51E−09	RI + slope	RI + slope	0.05 [− 0.08 to 0.18]	Yes	
	Natriuretic peptides B	1.77E−07	RI + slope	RI + slope	0.32 [0.19 to 0.43]	Elevated	FDA approved
	DNA-3-methyladenine glycosylase	2.44E−06	RI + slope	RI	0.00 [− 0.14 to 0.13]	Yes	
	AP-2 complex subunit alpha-2	4.58E−05	RI + slope	RI	0.10 [− 0.02 to 0.22]	Yes	
	Atrial natriuretic factor	4.40E−04	RI + splines	RI + slope	0.26 [0.14 to 0.38]	Elevated	FDA approved
	Ficolin-3	3.16E−03	RI + splines	RI + slope	− 0.14 [− 0.27 to 0.00]	Yes	
	Low density lipoprotein receptor adapter protein 1	1.34E−02	RI	RI	0.11 [− 0.02 to 0.24]	Yes	
	Phosphatidylinositol 4,5-bisphosphate 3-kinase catalytic subunit gamma isoform	3.43E−02	RI	RI	0.26 [0.14 to 0.38]	Yes	FDA approved
	Phospholipase A2, membrane associated	4.36E−02	RI + slope	RI	0.01 [− 0.12 to 0.13]	Yes	FDA approved
	Iron-sulfur cluster co-chaperone protein HscB, mitochondrial	4.62E−02	RI	RI	0.28 [0.17 to 0.40]	Yes	
EDV	Calcium-binding protein 39	2.21E−158	RI	RI	0.04 [− 0.09 to 0.16]	Yes	
	Histidine-tRNA ligase, cytoplasmic	6.86E−155	RI	RI	0.03 [− 0.1 to 0.15]	Yes	
	Ubiquitin carboxyl-terminal hydrolase 8	3.81E−154	RI	RI	0.04 [− 0.09 to 0.17]	Yes	Potential target
	Calpain I	6.44E−153	RI	RI	0.07 [− 0.06 to 0.19]	Yes	Potential target
	Poly(rC)-binding protein 1	1.96E−150	RI	RI	0.07 [− 0.06 to 0.19]	Yes	
	Synaptosomal-associated protein 25	4.16E−142	RI	RI	0.09 [− 0.03 to 0.21]	Yes	FDA approved
	Acetyl-CoA acetyltransferase, cytosolic	8.87E−140	RI	RI	0.1 [− 0.03 to 0.22]	Yes	
	Zinc transporter 3	1.84E−09	RI + slope	RI + splines	0.17 [0.05 to 0.29]	Yes	
	Lysyl oxidase homolog 3	3.52E−09	RI	RI + slope	0.07 [− 0.06 to 0.20]	Yes	Potential target
	Myosin light chain 3	1.63E−08	RI + slope	RI + splines	0.01 [− 0.12 to 0.13]	Elevated	
	BAG family molecular chaperone regulator 1	3.57E−08	RI + slope	RI + splines	0.06 [− 0.06 to 0.19]	Yes	
	G2/mitotic-specific cyclin-B2	7.01E−08	RI + slope	RI + splines	0.18 [0.05 to 0.30]	Not detected	
	Heterogeneous nuclear ribonucleoprotein A/B	7.27E−08	RI + slope	RI + splines	0.06 [− 0.05 to 0.18]	Yes	
	Protein phosphatase 1 A	7.77E−08	RI + slope	RI + splines	− 0.09 [− 0.22 to 0.04]	Yes	
	Serine/threonine-protein kinase 17B	1.08E−07	RI + slope	RI + splines	0.07 [− 0.06 to 0.19]	Yes	
	Chondrocalcin	1.37E−07	RI + slope	RI + splines	− 0.09 [− 0.2 to 0.04]	Not detected	FDA approved
	Fibroblast growth factor 7	1.51E−07	RI + slope	RI + splines	0.00 [− 0.12 to 0.12]	Yes	
	AP-2 complex subunit alpha-2	1.64E−07	RI + slope	RI + splines	0.04 [− 0.08 to 0.16]	Yes	
	CREB-binding protein	2.25E−07	RI + slope	RI + splines	0.00 [− 0.13 to 0.14]	Yes	Potential target
	Fibroleukin	3.75E−07	RI + slope	RI + splines	0.16 [0.02 to 0.29]	Yes	
	Axin interactor, dorsalization-associated protein	6.21E−07	RI + slope	RI + splines	0.03 [− 0.1 to 0.14]	Yes	
	Eukaryotic translation initiation factor 3 subunit J	8.34E−07	RI + slope	RI + splines	0.14 [0.01 to 0.26]	Yes	
	Vesicle transport through interaction with t-SNAREs homolog 1B	1.67E−05	RI	RI + slope	− 0.04 [− 0.18 to 0.09]	Yes	
	Natriuretic peptides B	4.53E−05	RI + slope	RI	0.15 [0.02 to 0.27]	Elevated	FDA approved
	N-terminal pro-BNP	1.37E−04	RI + slope	RI	0.18 [0.06 to 0.31]	Elevated	FDA approved
	Glia maturation factor beta	3.89E−04	RI + slope	RI + slope	− 0.01 [− 0.14 to 0.13]	Yes	
	Atrial natriuretic factor	9.03E−03	RI	RI + slope	0.11 [− 0.01 to 0.24]	Elevated	FDA approved
EF	Charged multivesicular body protein 3	3.30E−123	RI	RI	− 0.14 [− 0.26 to − 0.01]	Yes	
	Interleukin-1 receptor-associated kinase 4	1.75E−117	RI	RI	− 0.14 [− 0.26 to 0.00]	Yes	Potential target
	Carbonyl reductase [NADPH] 1	2.05E−112	RI	RI	− 0.09 [− 0.22 to 0.03]	Yes	
	Differentially yes in FDCP 6 homolog	1.64E−108	RI	RI	− 0.11 [− 0.24 to 0.02]	Yes	
	N-terminal pro-BNP	2.03E−14	RI + splines	RI + slope	− 0.33 [− 0.44 to − 0.21]	Elevated	FDA approved
	Natriuretic peptides B	3.79E−12	RI	RI + slope	− 0.30 [− 0.42 to − 0.19]	Elevated	FDA approved
	Angiopoietin-2	2.03E−02	RI + slope	RI + slope	− 0.24 [− 0.36 to − 0.11]	Yes	FDA approved
	Palmitoleoyl-protein carboxylesterase NOTUM	2.35E−02	RI + slope	RI + splines	0.15 [0.04 to 0.28]	Not detected	

FDR= false discovery rate; RI=random intercept; FDA=United States Food and Drug Administration. ^a^Spearman correlation between within patient means of echocardiographic variables and log-transformed protein levels. ^b^As defined by the Human Protein Atlas.

The mean values of the associated proteins were generally positively correlated with the mean values of AV and EDV, while they were generally negatively associated with mean values of LVEF. Exceptions were ficolin-3 and AV which were negatively associated, and palmitoleoyl-protein carboxylesterase (NOTUM) and LVEF which were positively associated. Most associated proteins are expressed in the heart, with four proteins showing elevated expression compared to other tissue types (NT-proBNP, BNP, ANP and myosin light chain 3). Three proteins did not show detectable expression in heart tissue, G2/mitotic-specific cyclin-B2, chondrocalcin and palmitoleoyl-protein carboxylesterase (NOTUM). Nine proteins were FDA (United States Food and Drug Administration) approved drug targets, while an additional five were designated as potential targets by the Human Protein Atlas.^12^.

Supplemental Table 3 displays the results of the enrichment analysis. Due to the smaller numbers of proteins in the sets these findings should be interpreted with care. There were eight significantly associated biological mechanisms with three or more associated proteins in the set, all for AV. These were related to heart contraction, inflammation and the organophosphate metabolic process.

## Discussion

In this study, we explored the plasma proteomic profile of post-MI patients, in order to gain insights into the pathophysiological processes that are associated with the evolvement of cardiac function. We focused on three echocardiographic variables associated with different aspects of cardiac function, including myocardial contractility, ventricular and atrial remodeling and pressure and volume load.

### Left-ventricular end-diastolic volume

We found 28 proteins with trajectories associated with change in LVEDV. Some of these can be directly related to cardiac contraction, such as myosin essential light chain (MYL3),^15^ and cardiac stress, such as NT-proBNP as well as BNP. Most others can be linked to mechanisms of cardiac remodeling, vascular dysfunction, and oxidative stress.

As for mechanisms of cardiac remodeling, lysyl oxidase homolog 3 (LOXL3) is upregulated in stressed hearts and plays a role in cardiac function and remodeling.^16,17^ Deficiency in calpain-1 catalytic subunit (CAPN1) has been shown to reduce remodeling after MI.^18^ Collagen, type II, alpha 1 (COL2A1) is involved in fibrotic scar formation after MI.^19^

Regarding vascular dysfunction, poly(rC)-binding protein 1 (PCBP1)^20^, histidyl-tRNA synthetase (HARS)^21^, and ubiquitin carboxyl-terminal hydrolase 8 (USP8)^22^ can be associated with (pulmonary) vascular development and remodeling. AP-2 complex subunit alpha-2 (AP2A2)^23^ and acetyl-CoA acetyltransferase (ACAT2)^24^ are related to CAD, while calcium-binding protein 39 (CAB39)^25^, heterogeneous nuclear ribonucleoprotein A/B (HNRNPAB)^26^ and cyclin B2 (CCNB2)^27^ are linked to calcific aortic valve disease, vascular smooth muscle cell function and cardiac endothelial cell proliferation, respectively.

Finally, keratinocyte growth factor (FGF7) plays a role in alleviating oxidative stress after MI^28^, and zinc transporter 3 (SLC30A3)^29^ plays an important role against oxidative and ER stress. BAG family molecular chaperone regulator 1 (BAG1) induces autophagy for cardiac cell survival after oxidative stress.^30^.

### Left atrial volume

For temporal evolvement of atrial volume, we found 12 proteins with related trajectories, among which cardiac stress-related NT-proBNP and BNP. The other proteins are mainly related to CAD and atherosclerosis, such as AP-2 complex subunit alpha-2 (AP2A2)^23^, low-density lipoprotein receptor adapter protein 1 (LDLRAP1)^31^, phospholipase A2 (PLA2G2A; membrane associated)^32^ and lysosomal alpha-glucosidase (GAA)^33^, as well as the immune system: phosphatidylinositol 4,5-bisphosphate 3-kinase catalytic subunit gamma isoform (PIK3CG)^34^ and ficolin-3 (FCN3)^35^.

### Left-ventricular ejection fraction

We found 8 proteins with temporal trajectories that were associated with the evolution of LVEF. Foremost again, NT-proBNP and BNP. Moreover, differentially expressed in FDCP 6 homolog (DEF6), which is associated with cardiac hypertrophy.^36^ Palmitoleoyl-protein carboxylesterase (NOTUM) is protective against MI-induced heart dysfunction by alleviating cardiac fibrosis.^37^ Furthermore, interleukin-1 receptor-associated kinase 4 (IRAK4) and angiopoietin-2 (ANGPT2) are related to cardiac inflammation.^38,39^.

### Summary of mechanisms

**Fig. 4:**
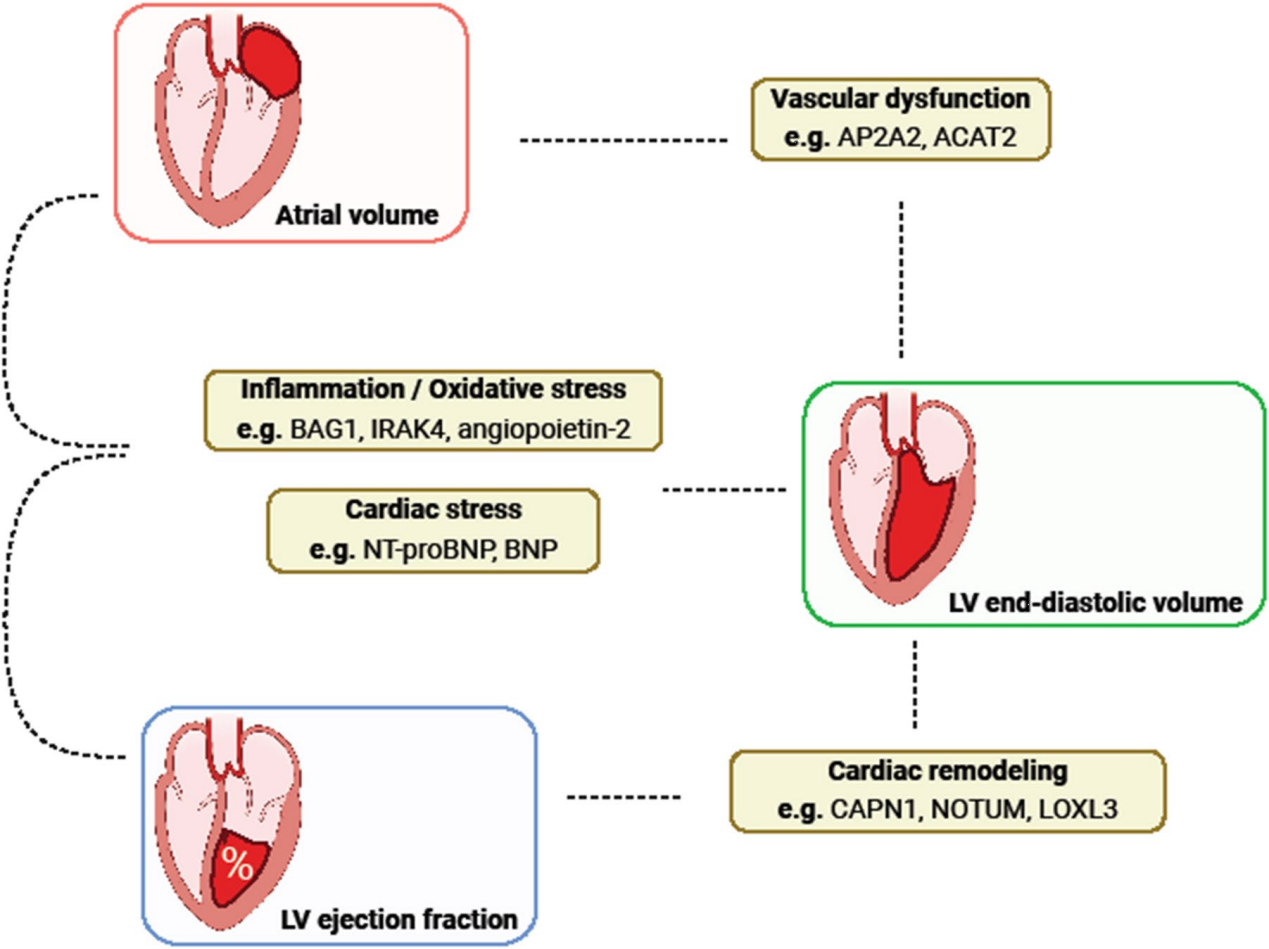
Summary of putative biological mechanisms related to evolution of echocardiographic variables as described in the discussion section.

Altogether, next to cardiac stress, our results implicated three main categories of mechanisms associated with cardiac function after MI, namely cardiac remodeling, vascular dysfunction and inflammation or oxidative stress, as illustrated in Fig. 4. The heart is known to undergo extensive remodeling after MI due to the accumulation of fibrous tissue.^40^ This process can increase stiffness and impair normal heart relaxation which can have an adverse effect on the efficiency and function of the heart. It is therefore not surprising that we found plasma proteins representing such mechanisms to be related with LVEDV and LVEF. Furthermore, many proteins that were found to be associated with LVEDV and AV were related to vascular development, atherosclerosis, and CAD. CAD, in addition to being the primary cause of MI, can impair myocardial contractility, which may lead to HF.^41^ Vascular dysfunction has been associated with increased left atrial pressure, which can cause increasing AV.^42^ Finally, associations with proteins related to inflammation and oxidative stress were present for all three echocardiographic measures. Inflammation plays an intricate role in the pathophysiology of HF as it could both be its cause and effect.^43^ Differences in inflammatory response to acute MI have been implicated to affect LV remodeling.^44^ Its association with all three echocardiographic measures emphasizes its ubiquitous involvement in progression of cardiac function following MI.

### Translational outlook

Several of the proteins found to be associated with adverse functional changes have been designated as potential or approved druggable targets. After further study, these could inspire novel therapeutic strategies to manage the deterioration of cardiac function after MI. For example, this could include targeting inflammatory pathways involving proteins like interleukin-1 receptor-associated kinase 4 (IRAK4) or pathways of cardiac remodeling involving lysyl oxidase homolog 3 (LOXL3). However, external validation is warranted due to the hypothesis generating nature of this study.

Furthermore, these findings could contribute to the development of sophisticated multiplex plasma biomarker panels for post-MI patients. While NT-proBNP and BNP are established markers of cardiac stress, a broader panel incorporating proteins linked to remodeling, vascular health, and inflammation could allow for more precise risk stratification of post-MI patients. This could help clinicians to identify individuals at high risk for developing HF and to tailor preventative treatments to the putative specific underlying pathophysiological mechanisms.

### Strengths and limitations

This study is a first in its kind to explore proteomic biomarkers and pathways implicated in the evolution of LV and atrial function after MI. We utilize a relatively large and broad panel of circulating proteomic biomarkers to account for the heterogeneous nature of cardiac dysfunction. Moreover, using multiple serial measurements of both the measured proteins and the echocardiograms allows us to follow LV and atrial function, and related mechanisms dynamically.

Limitations of our study include the large proportion of men (81%) and the fact that the population suffered from severe MI. Furthermore, while most patients had acute reperfusion, the relatively low proportion of patients with primary PCI reflects the practice in 2006–2008, and was lower than it would be nowadays. Due to concerns surrounding statistical power we did not include subanalyses stratified by treatment. Investigating these potential differences in a larger cohort represents a key opportunity for future research. Moreover, while most of the circulating proteins we found are expressed in heart tissue, most are not cardiac specific, so they may not necessarily reflect what happens on a cardiac tissue level. Finally, plasma samples were collected without the addition of protease inhibitors. While we adhered to standard protocols using EDTA plasma, which is consistent with large-scale proteomic studies, the potential for proteolysis of susceptible proteins prior to analysis cannot be excluded.

## Conclusions

To conclude, we identified various proteomic biomarkers and mechanisms related to the evolution of cardiac function in patients with recent MI. Our results give an overview of the most important mechanisms related to the deterioration of cardiac function after MI. Overall, this study underscores the multifaceted pathophysiology of post-MI loss of cardiac function, with cardiac stress, cardiac remodeling, vascular dysfunction, and inflammation/oxidative stress emerging as central mechanisms.

## Supplementary Information

Below is the link to the electronic supplementary material.

Supplementary Material 1

## Data Availability

Anonymized data that support the findings of this study will be made available to other researchers for the purposes of reproducing the results in accordance with a data-sharing agreement, upon reasonable request to Dr. Florence Pinet.
