# Supplementary material for: Plasma proteins and mechanisms involved in the evolvement of cardiac function after myocardial infarction
Source: Sci Rep. 2026 Mar 12;16:13251. doi: 10.1038/s41598-026-43659-6 (PMC13103015; doi:10.1038/s41598-026-43659-6)
Supplement: Supplementary file 1 — Supplementary Material 1 [file 41598_2026_43659_MOESM1_ESM.docx]

Supplemental Material:
Plasma proteins and mechanisms involved in the evolvement of cardiac function after myocardial infarction

Teun B. Petersen^a,b,c^; Dimitris Rizopoulos^b,c^; Eric Boersma^a^; Florence Pinet^d^; Isabella Kardys^a^*; Christophe Bauters^d^*

a Department of Cardiology, Thorax Center, Cardiovascular Institute, Erasmus MC, Rotterdam, the Netherlands.

b Department of Biostatistics, Erasmus MC, University Medical Center Rotterdam, Rotterdam, the Netherlands.

c Department of Epidemiology, Erasmus MC, University Medical Center Rotterdam, Rotterdam, the Netherlands.

d Inserm, CHU Lille, Institut Pasteur de Lille, U1167- RID-AGE, Université de Lille, Lille, France.

* These authors contributed equally

**Address for correspondence:** Isabella Kardys, MD, PhD, Department of Cardiology, Erasmus MC, University Medical Center Rotterdam, Room Na‐316, P.O. Box 2040, 3000 CA Rotterdam, the Netherlands. E‐mail: i.kardys@erasmusmc.nl Phone: +31650032051

**Supplemental Methods**

**Considerations on statistical power**

The REVE-2 study was initially powered to detect a significant difference in B-type natriuretic peptide (BNP) levels between patients with and without left ventricular (LV) remodeling. The sample size calculation was based on detecting a 30% difference in BNP levels at a two-sided significance alpha level of 0.05. This calculation indicated that a sample of 200 patients would be sufficient for a power exceeding 80%, and the final enrolled cohort of 246 patients exceeded this requirement.^1^

The current analysis involved finding the association between 4587 proteins and echocardiographic variables. To provide a conservative estimate of the power, we can consider a simplified cross-sectional scenario. If we aim to detect a correlation of -0.32 (similar to the correlation of mean NT-proBNP and mean LVEF in the REVE-2 study) while employing a Bonferroni corrected alpha of 1.1E-5 (0.05/4587), we find that our sample size of 246 patients achieves a statistical power of approximately 82.2%. However, we expect that actual power is substantially higher, as our main analysis employed a longitudinal design with serial measurements (which increase power), and utilized the Benjamini-Hochberg procedure to account for multiple testing.

**Supplemental Table 1: Availability of measurements at baseline and after 1, 3, and 12 months**

|  | **Baseline** | **1 month** | **3 months** | **12 months** |
| --- | --- | --- | --- | --- |
| **Echocardiogram** | 236 (96%) | - | 216 (88%) | 220 (89%) |
| **Circulating proteins** | - | 232 (94%) | 224 (91%) | 224 (91%) |

**Supplemental Table 2:** Random effect structure multivariate linear mixed effect models

|  | **Left atrial volume** | | | | |
| --- | --- | --- | --- | --- | --- |
| **Protein** |  | **did not converge** | **random intercept** | **random intercept**  **+ slope** | **random intercept**  **+ splines** |
|  | **did not converge** | 159 | 0 | 0 | 0 |
|  | **random intercept** | 0 | 545 | 210 | 0 |
|  | **random intercept**  **+ slope** | 0 | 1044 | 1046 | 537 |
|  | **random intercept**  **+ splines** | 0 | 0 | 533 | 760 |

|  | **Left-ventricular ejection fraction** | | | | |
| --- | --- | --- | --- | --- | --- |
| **Protein** |  | **did not converge** | **random intercept** | **random intercept**  **+ slope** | **random intercept**  **+ splines** |
|  | **did not converge** | 186 | 0 | 0 | 0 |
|  | **random intercept** | 0 | 338 | 220 | 0 |
|  | **random intercept**  **+ slope** | 0 | 713 | 719 | 1176 |
|  | **random intercept**  **+ splines** | 0 | 0 | 342 | 1140 |

|  | **Left-ventricular end-diastolic volume** | | | | |
| --- | --- | --- | --- | --- | --- |
| **Protein** |  | **did not converge** | **random intercept** | **random intercept**  **+ slope** | **random intercept**  **+ splines** |
|  | **did not converge** | 126 | 0 | 0 | 0 |
|  | **random intercept** | 0 | 228 | 313 | 0 |
|  | **random intercept**  **+ slope** | 0 | 331 | 995 | 760 |
|  | **random intercept**  **+ splines** | 0 | 0 | 383 | 1698 |

**Supplemental Table 3:** Gene Ontology: biological mechanisms associated with AV- EDV- and EF-related proteins

|  | **ID** | **Name** | **FDR** | **Hit Count Set** | **Hit Count in SomaScan Panel** | **Hit in Set** |
| --- | --- | --- | --- | --- | --- | --- |
| **AV** | GO:0006182 | cGMP biosynthetic process | 8,42E-04 | 2 | 2 | NPPA,NPPB |
|  | GO:1903814 | regulation of collecting lymphatic vessel constriction | 8,42E-04 | 2 | 2 | NPPA,NPPB |
|  | GO:1990183 | lymphatic vascular process in circulatory system | 8,42E-04 | 2 | 2 | NPPA,NPPB |
|  | GO:1990186 | regulation of lymphatic vessel size | 8,42E-04 | 2 | 2 | NPPA,NPPB |
|  | GO:1990192 | collecting lymphatic vessel constriction | 8,42E-04 | 2 | 2 | NPPA,NPPB |
|  | GO:0007168 | receptor guanylyl cyclase signaling pathway | 8,42E-04 | 2 | 2 | NPPA,NPPB |
|  | GO:0010753 | positive regulation of cGMP-mediated signaling | 1,89E-03 | 2 | 3 | NPPA,NPPB |
|  | GO:0009190 | cyclic nucleotide biosynthetic process | 1,89E-03 | 2 | 3 | NPPA,NPPB |
|  | GO:0010752 | regulation of cGMP-mediated signaling | 5,59E-03 | 2 | 5 | NPPA,NPPB |
|  | GO:0046068 | cGMP metabolic process | 7,54E-03 | 2 | 6 | NPPA,NPPB |
|  | GO:0060048 | cardiac muscle contraction | 1,61E-02 | 3 | 50 | GAA,NPPA,PIK3CG |
|  | GO:0014898 | cardiac muscle hypertrophy in response to stress | 1,61E-02 | 2 | 10 | NPPA,NPPB |
|  | GO:0014887 | cardiac muscle adaptation | 1,61E-02 | 2 | 10 | NPPA,NPPB |
|  | GO:0003299 | muscle hypertrophy in response to stress | 1,61E-02 | 2 | 10 | NPPA,NPPB |
|  | GO:0019934 | cGMP-mediated signaling | 1,83E-02 | 2 | 11 | NPPA,NPPB |
|  | GO:0006941 | striated muscle contraction | 1,97E-02 | 3 | 59 | GAA,NPPA,PIK3CG |
|  | GO:0003012 | muscle system process | 2,74E-02 | 4 | 168 | GAA,NPPA,PIK3CG,NPPB |
|  | GO:0052652 | cyclic purine nucleotide metabolic process | 2,74E-02 | 2 | 15 | NPPA,NPPB |
|  | GO:0003085 | negative regulation of systemic arterial blood pressure | 2,74E-02 | 2 | 15 | NPPA,NPPB |
|  | GO:0003161 | cardiac conduction system development | 2,83E-02 | 2 | 16 | NPPA,NPPB |
|  | GO:0009187 | cyclic nucleotide metabolic process | 2,83E-02 | 2 | 16 | NPPA,NPPB |
|  | GO:0014888 | striated muscle adaptation | 3,36E-02 | 2 | 18 | NPPA,NPPB |
|  | GO:0008016 | regulation of heart contraction | 3,36E-02 | 3 | 80 | GAA,NPPA,PIK3CG |
|  | GO:0019637 | organophosphate metabolic process | 4,48E-02 | 5 | 366 | PLA2G2A,DLG2,NPPA,PIK3CG,NPPB |
|  | GO:0060047 | heart contraction | 4,79E-02 | 3 | 93 | GAA,NPPA,PIK3CG |
|  | GO:0050729 | positive regulation of inflammatory response | 4,90E-02 | 3 | 95 | PLA2G2A,NPPA,PIK3CG |
|  | GO:0042311 | vasodilation | 4,98E-02 | 2 | 24 | NPPA,NPPB |
|  | GO:0003015 | heart process | 4,98E-02 | 3 | 98 | GAA,NPPA,PIK3CG |
| **EDV** | GO:0006182 | cGMP biosynthetic process | 7,21E-03 | 2 | 2 | NPPA,NPPB |
|  | GO:1903814 | regulation of collecting lymphatic vessel constriction | 7,21E-03 | 2 | 2 | NPPA,NPPB |
|  | GO:1990183 | lymphatic vascular process in circulatory system | 7,21E-03 | 2 | 2 | NPPA,NPPB |
|  | GO:1990186 | regulation of lymphatic vessel size | 7,21E-03 | 2 | 2 | NPPA,NPPB |
|  | GO:1990192 | collecting lymphatic vessel constriction | 7,21E-03 | 2 | 2 | NPPA,NPPB |
|  | GO:0007168 | receptor guanylyl cyclase signaling pathway | 7,21E-03 | 2 | 2 | NPPA,NPPB |
|  | GO:0010753 | positive regulation of cGMP-mediated signaling | 1,62E-02 | 2 | 3 | NPPA,NPPB |
|  | GO:0009190 | cyclic nucleotide biosynthetic process | 1,62E-02 | 2 | 3 | NPPA,NPPB |
|  | GO:0010752 | regulation of cGMP-mediated signaling | 4,76E-02 | 2 | 5 | NPPA,NPPB |
| **EF** | None | | | | | |

FDR= False discovery rate

**Supplemental references**

1. Fertin M, Hennache B, Hamon M, Ennezat PV, Biausque F, Elkohen M, et al. Usefulness of serial assessment of B-type natriuretic peptide, troponin I, and C-reactive protein to predict left ventricular remodeling after acute myocardial infarction (from the REVE-2 study). The American journal of cardiology. 2010;106(10):1410-6.
